# Jugular foramen and venous collaterals may help to discriminate congenital from post-thrombotic jugular stenosis

**DOI:** 10.1186/s40001-022-00636-9

**Published:** 2022-01-13

**Authors:** Xiaoqin Wu, Yuchuan Ding, Xunming Ji, Ran Meng

**Affiliations:** 1 Department of Neurology, Xuanwu Hospital, Capital Medical University, Beijing, 100053 China; 2 Advanced Center of Stroke, Beijing Institute for Brain Disorders, Beijing, 100053 China; 3 Department of China-America Institute of Neuroscience, Xuanwu Hospital, Capital Medical University, Beijing, 100053 China; 4 Department of Neurosurgery, Wayne State University School of Medicine, Detroit, MI 48201 USA

**Keywords:** Cerebral venous thrombosis, Acquired jugular stenosis, Jugular hypoplasia, Jugular foramen, Venous collaterals, Case report

## Abstract

**Purpose:**

Unilateral jugular stenosis is easily mistaken as jugular hypoplasia for their similar jugular appearances. This study aimed to propose a scheme to differentiate acquired internal jugular vein stenosis (IJVS) from congenital jugular variation through two case examples.

**Methods:**

We presented a dynamic evolution process of the IJVS formation, through a case of a 17-year-old female with paroxysmal nocturnal hemoglobinuria (PNH)-associated right internal jugular venous thrombosis (IJVT), which resulted in post-thrombotic IJVS in the rare context of rapid recanalization. Meanwhile, we compared her images with images of a 39-year-old healthy male with hypoplastic IJV to determine the differences between the acquired IJVS and congenital dysplasia.

**Results:**

Based on the first case, we noticed the whole formative process of acquired IJVS from nothing to something. Meantime, we found that acquired IJVS was surrounded by abnormal corkscrew collaterals as imaged on contrast-enhanced magnetic resonance venography (CE-MRV), and the ipsilateral jugular foramen (JF) was normal-sized as displayed on computer tomography (CT). Conversely, jugular hypoplasia was with ipsilateral stenotic JF and without serpentine collaterals.

**Conclusion:**

JF morphology and venous collaterals may be deemed as surrogate identifiers to distinguish acquired unilateral IJVS from jugular hypoplasia.

## Background

When faced with unilateral internal jugular vein stenosis (IJVS), we are often confused about whether the slender side was anatomically dysplasia or acquired stenosis. For which, previous study revealed that patients with acquired IJVS likely had serpiginous collaterals around the involved IJV, which may serve as the imaging evidence to differentiate it from jugular hypoplasia [1, 2]. Herein, we displayed a first novel report regarding the dynamic formation of the post-thrombotic IJVS on the rare occasion of rapid IJV thrombosis (IJVT) resolution with excellent patency, and therefore built up a comprehensive scheme to ascertain the origins of IJVS. Besides, despite paroxysmal nocturnal hemoglobinuria (PNH)-triggered thrombosis is a well-described condition, non-thrombotic IJVS develops as a sequela of PNH-associated IJVT as described in current study has never been reported previously.

## Case presentation

**Fig. 1:**
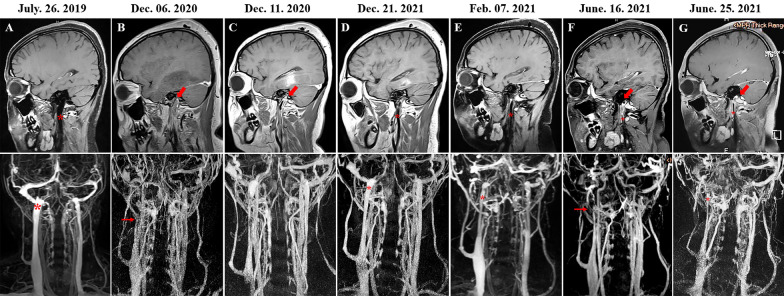
Magnetic resonance black-blood thrombus imaging (MRBTI) and contrast-enhanced MR venography (CE-MRV). **A** The thrombi located within cerebral venous sinus without internal jugular vein (IJV) involvement. The right IJV (red asterisk) was clearly visualized. **B** The recurrent thrombi blocked the upper segments of right IJV (red arrow) and the right IJV (red thin arrow) was invisible surrounded by abnormal collaterals. **C** The right IJV thrombosis (IJVT) with partial recanalization after mechanical venous thrombectomy. **D**, **E** The narrowed jugular lumen (red asterisk) after the complete recovery of IJVT. **F** The relapsing right IJVT (red arrow) and the right IJV opacification (red thin arrow). **G** The almost completely recanalized IJVT (red arrow) and the much more narrowed right jugular lumen

**Fig. 2:**
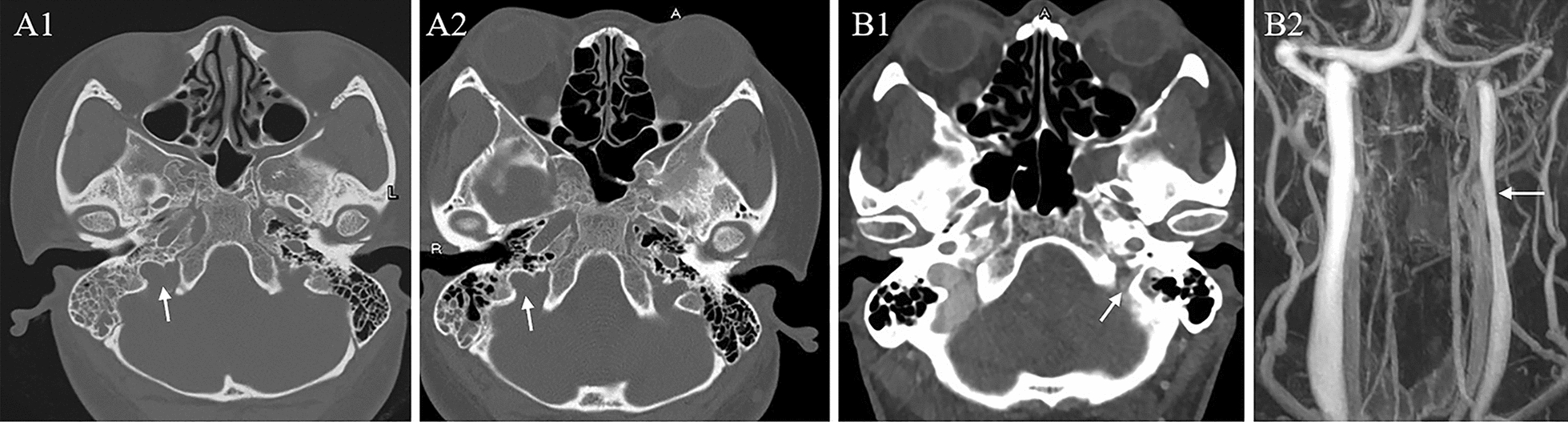
Initial and follow-up computed tomography (CT) maps, and a representative case with jugular hypoplasia. Right jugular foramen (JF, white thin arrow) on baseline CT image (**A1**) appeared to be the same as that on 2-year CT follow-up (**A2**). A 39-year-old male for physical screening was imaged with narrowed ipsilateral JF (**B1**, white thin arrow) along with hypoplastic IJV (**B2**, white thin arrow) on CE-MRV

A 17-year-old female was hospitalized for severe pains in her head and neck for 1 week. She had an episode of 1-year poorly controlled PNH prior to this admission. This patient denied history of lower-limb deep venous thrombosis, sexual activity and oral contraception use. Other cerebral venous thrombosis (CVT) hazards were also excluded according to the results of routine inpatient check-ups. Positive physical signs included moderate nuchal stiffness and pallor of complexion and conjunctiva. Blood tests indicated severe anemia with hemoglobin concentration of 5.6 g/dL and red blood cell (RBC) count of 1.76 × 10^12^/L. Baseline lumbar puncture opening pressure (LPOP) was above 330 mmH_2_O. Magnetic resonance black-blood thrombus imaging (MRBTI) and contrast-enhanced MR venography (CE-MRV) confirmed CVT with bilateral unaffected IJVs at that moment (Fig. 1A). Aside from optimal PNH management and washed RBC transfusion for anemia correction, this patient underwent anticoagulant plus dehydrated treatment, and ultimately obtained clinical relief with LPOP decreasing to 215 mmH_2_O. However, 16 months later, her symptoms relapsed with hemoglobin declining to a critical value of 4.8 g/dL. Repeated neuroimaging confirmed recurrent CVT propagating to the upper segment of right IJV (Fig. 1B). She hence underwent blood re-transfusion followed by immediate mechanical thrombectomy and continuous anticoagulation. Afterwards, the thrombus-blocked IJV obtained recanalization step by step (Fig. 1C–E). However, we observed that her inflicted jugular lumen was obviously slimmer than that before the course of IJVT and surrounded by tortuous collaterals, despite total patency (Fig. 1D, E). Unfortunately, her symptoms reappeared twice again half year later with neuroimaging-verified thrombus recurrence (Fig. 1F). For which, she was treated with anticoagulation to derive both clinical and imaging remission; nevertheless, her right-side IJV seemed progressively slenderer than the previous one (Fig. 1G). Such jugular narrowing was the acquired deformation with abnormally dilated collaterals, but may possibly mimic primary dysplasia. Despite the obvious tapering of jugular conduit, her right normal-sized jugular foramen (JF) remained unvarying on the computed tomography (CT) maps at both baseline (Fig. 2-A1) and 2-year follow-up (Fig. 2-A2). Actually, the ipsilateral JF should be stenotic and no anomalous collaterals created in cases with jugular hypoplasia, as illustrated by the images of another 39-year-old male, who was admitted for routine physical examination and identified with jugular hypoplasia (Fig. 2-B1, B2).

## Discussion

Several highlights of this study can be summarized as follows: Firstly, this study detailed an evolutionary process regarding the formation of post-thrombotic IJVS based on a case with PNH-associated right jugular thrombosis. Additionally, this study perceived that acquired IJVS might impossibly make the dimension of JF shrink to be narrowing based on a longer than 2-year follow-up. Finally, through comparing the images to another case with jugular variation, we hence uncovered the differences between these two different entities and thus proposed a method about how to determine the source of unilateral IJVS.

Considering venous stenosis is a general postlude of deep venous thrombosis [3], post-thrombotic IJVS described in this study is likely a widely ignored universal rather than an epiphenomenon. However, it might be misinterpreted as jugular hypoplasia in clinical settings. Recent studies confirmed the post-thrombotic venous wall injury to be one of the culprits of IJVS and the string collaterals may possibly develop to share the drainage burden of the thrombosed IJV [1, 3]. Based on which, our findings further disclosed that the size of JF and the presence of collateral varicosity might function to distinct primary and acquired IJVS. Moreover, given the nature of case reports, we failed to confirm that the dimension of JF was unlikely to remodel or occlusion in the setting of acquired IJVS for the lack of conclusion reliability. Additionally, our result discorded with an artery study that exhibiting degenerative carotid canal followed with carotid artery occlusion over 6-year follow-up [4]. Whereby, longer-term follow-up with large sample sizes is still warranted in the future.

## Conclusion

To sum up, non-thrombotic IJVS may emerge as a residual stenosis of post-IJVT, even with rapid resolution and complete patency. JF size and IJV collaterals may function as the imaging signage to differentiate post-thrombotic jugular stenosis from congenital jugular dysplasia.

## Data Availability

Data and materials related to the current study are available from our corresponding author upon reasonable request.
